# Periostin Expression is Altered in Aortic Valves in Smad6 Mutant Mice

**DOI:** 10.4172/2167-0897.1000101

**Published:** 2012-01-21

**Authors:** Yukiko Sugi, Michael J Kern, Roger R Markwald, Jessica L Burnside

**Affiliations:** Department of Regenerative Medicine and Cell Biology, Cardiovascular Developmental Biology Center, Medical University of South Carolina, USA

**Keywords:** Periostin, Versican, Smad6, Hyaluronan, Heart valve, Outflow tract, Endocardial cushion

## Abstract

Smad6 is known to predominantly inhibit BMP signaling by negatively regulating the BMP signaling process. Therefore, Smad6 mutation potentially provides an important genetic model for investigating the role of BMP signaling in vivo. Periostin is a 90-kDA secreted extracellular matrix (ECM) protein and implicated in cardiac valve progenitor cell differentiation, maturation and adult aortic valve calcification in mice. We have previously reported periostin expression patterns during AV valve development in mice. Because periostin can play critical roles in aortic valve interstitial cell differentiation and can be correlated with adult valve disease pathogenesis, in the present study we specifically focused on periostin expression during outflow tract (OT) development and its expression within the adult mouse valves. We previously reported that periostin expression in valve progenitor cells was altered by exogenously adding BMP-2 in culture. In this study, we investigated whether expression of periostin and other valvulogenic ECM proteins was altered in Smad6-mutant newborn mice in vivo. Periostin protein was localized within OT during embryonic development in mice. At embryonic day (ED) 13.5, robust periostin expression was detected within the developing pulmonary trunk and developing pulmonary and aortic valves. Periostin expression remained intense in pulmonary and aortic valves up to the adult stage. Our immunohistochemical and immunointensity analyses revealed that periostin expression was significantly reduced in the aortic valves in Smad6−/− neonatal hearts. Versican expression was also significantly reduced in Smad6−/− aortic valves, whereas, hyaluronan deposition was not significantly altered in the Smad6−/− neonatal valves. Expression of periostin and versican was less prominently affected in AV valves compared to the aortic valves, suggesting that a cell lineage/origin-dependent response to regulatory molecules may play a critical role in valve interstitial cell development and ECM protein expression.

## Introduction

Defects in valvuloseptal morphogenesis are among the most common and serious of all congenital heart defects [1, 2]. About one-fourth of patients with congenital heart disease have structural anomalies of one or more heart valves [3, 4]. During cardiac development, two segments of the endocardium-atrioventricular (AV) and outflow tract (OT)-transform into the cushion mesenchyme, primordia of the valves and membranous septa via epithelial mesenchymal transformation (EMT). Despite the fact that many genes and molecules are identified to play critical roles in EMT [5–7], we have limited understanding of how the cushion mesenchyme, after-EMT, subsequently differentiates and remodels into functional valves.

Bone morphogenetic protein (BMP) is a member of the transforming growth factor (TGFβ) superfamily proteins. BMP exerts its biological functions by interacting with cell surface receptors, Type I and Type II BMP receptors [8]. BMP signaling, among many other molecules, is found essential for AV EMT in studies with explants cultures in mouse and chick [9, 10] and in conditional knockout (CKO) experiments in mice [11–14]. Furthermore, we have shown that BMP-2 induces cell migration and periostin expression in endocardial cushion mesenchymal cells during AV valvulogenesis after EMT [15].

BMP signaling is regulated through Smad transcription factors. In particular, Smad6 has been shown to inhibit BMP signaling by negatively regulating the BMP signaling process via binding to a BMP receptor subunit or Smad 1/5/8 [16]. Smad6 is localized in the OT and AV prevalvular cushions of mouse [17] and chick [18]. Null ablation of the Smad6 results in hyperplasia of the cardiac outflow tract (OT) valves and cartilaginous metaplasia in the aortic wall [17]. Therefore, Smad6-mutant mice, in which BMP signaling can be constitutively elevated, provide an important genetic model for investigating the role of BMP signaling during valvulogenesis.

Periostin is a 90-kDA secreted extracellular matrix (ECM) protein, related to the midline fasciclin-1 gene in Drosophila [19] and has been identified as a heart enriched gene [20]. Periostin is highly expressed in cardiac cushions [20, 21] and implicated in valve primordial differentiation and maturation [22–24]. Specifically we found that periostin regulates collagen fibrillogenesis [25] and enhances cell invasion/migration of valve primordial cushion mesenchymal cells and collagen compaction [26]. More strikingly, periostin–null mice exhibit atrial septal defects and structural defects in AV valves and their supportive apparatus [27]. We have previously reported periostin expression patterns focusing on AV valve development and early stages of valvulogenesis [27]; however, periostin expression in OT in later development and in the adult has not been rigorously addressed.

As we reported previously, BMP signaling regulates periostin expression during AV valve endocardial cushion cell differentiation and maturation [15]. However, the role of BMP signaling in later stage valvulogenesis has not been addressed *in vivo* in regulating periostin and other critical valvulogenic ECM protein expression. Therefore, by taking advantage of the Smad6-mutation which may provide an important genetic model for investigating the role of constitutively up-regulated BMP signaling, in this article we present the expression pattern of periostin and other valvulogenic ECM proteins, focusing on later stage valvulogenesis. We compare their expression patterns in Smad6−/− and –wild type neonatal mouse hearts.

## Materials and Methods

### Immunohistochemical localization of periostin in outflow tract in mouse cardiac development

Preparation and characterization of affinity purified anti-mouse periostin antibodies were described previously [28]. Immunohistochemical procedures for mouse embryos and adult hearts were also described previously [9, 27]. Briefly, mouse embryos from ED 9.0-ED 13.5, hearts from mouse fetuses at ED16 and adults were collected and fixed with cold 100% methanol. Fixed samples were processed through descending methanol series and embedded in paraffin. Serial sections were cut, deparaffinized and blocked in 10% normal goat serum (MP Biomedicals) in 1% bovine albumin (BSA, Sigma)/phosphate buffered saline (PBS, pH 7.4) and processed for double immunohistochemistry. Sections were incubated with anti-mouse periostin antibodies (3ng/ml) followed by FITC-labeled goat anti-rabbit IgG (MP Biomedicals) treatments. Normal rabbit IgG was used as a negative control for anti-mouse periostin antibodies. Sections were counter immunostained with a myocardial marker, MF20 (Developmental Studies Hybridoma Bank) followed by RITC-labeled goat anti mouse-IgG (MP Biomedicals). Immunostained sections were examined under a Leica BMLB fluorescent microscope.

### Smad6 mutant mice and immunohistochemical localization of periostin, versican aggrecan and hyaluronan (HA) in neonatal mouse hearts

Smad6 mutant mice were originally provided by Dr. Galvin (Millennium Pharmaceuticals). Generation of Smad6 mutant mice is described in Galvin et al. [17]. PCR-genotyping was performed to identify Smad6 mutant embryos and newborn mice.

Smad6−/− (null) and Smad6+/+ (wild type) newborn mice were fixed in 4% paraformaldehyde in PBS and dissected mouse hearts were processed for paraffin embedding. Serial sections were cut and immunohistochemistry was performed for localizing periostin, versican, aggrecan and hyaluronan. Anti-aggrecan and anti-versican antibodies were purchased from Chemicon and used at the final concentration of 10 ng/ml. The immunohistochemical localization procedure was essentially the same as described above. HA was localized by using biotinylated hyaluronic acid binding protein (bHABP) obtained from Northstar Associates and following the procedure described previously [29]. Immunostained sections were examined under a Leica BMLB fluorescent microscope.

### Immunointensity analysis for quantitative assessment of the results

Immunointensity analysis was performed as described in Inai et al. [15]. Briefly, the immunointensity of periostin, versican and hyaluronan was evaluated by measuring the intensity of the immunofluorescence of the valves on photographs using computer software, Adobe Photoshop, CS2. Samples were collected from three Smad6−/− and four wild type neonatal mouse hearts. Three sections representing the middle parts of the valves were selected from each heart for Immunointensity statistical analysis.

### Statistical analysis

Student’s t-test was used to compare groups. Findings are represented as mean with standard errors of the mean. P<0.05 was considered significant.

## Results

### Periostin is intensely expressed in the valve primordia during OT valvulogenesis and in adult

**Figure 1:**
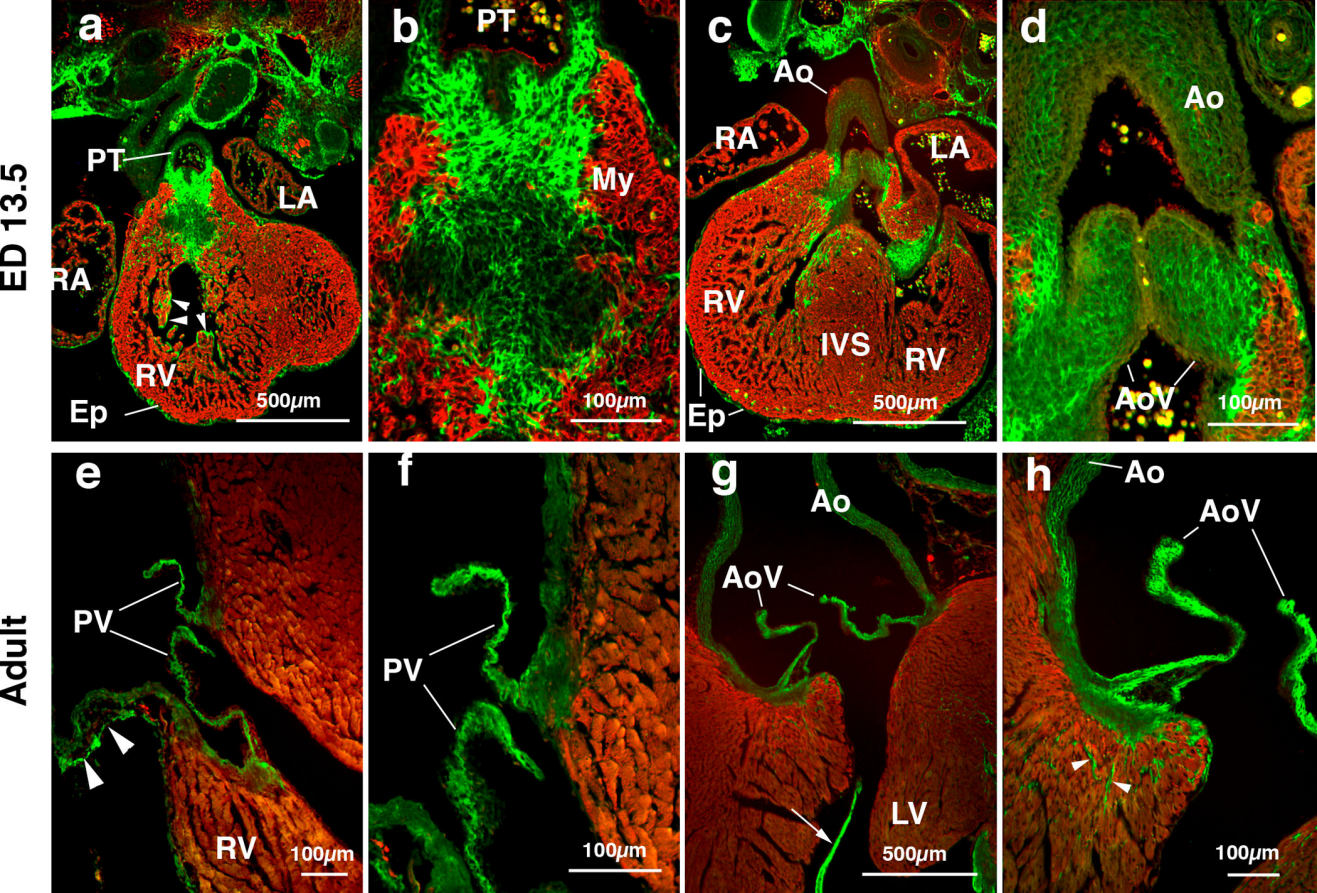
Periostin localization in mouse cardiac outflow tract (OT) and OT valves at embryonic day (ED) 13.5 (panels a-d) and in adult (panels e-h). (a) Frontal section of an ED 13.5 mouse embryo shows robust expression of periostin (green) in the pulmonary trunk (PT). Epicardium (Ep) and endocardial lining of the ventricular trabeculae (arrow heads) show intense periostin expression. MF20 staining is confined to the myocardium (My, red). (b) Higher magnification view of the pulmonary trunk mesenchyme in panel a, showing fibrous immunostaining of periostin. (c) Frontal section of an ED 13.5 mouse embryo shows intense expression of periostin in the forming aortic valves. (d) Higher magnification view of the forming aorta in panel c, showing intense expression of periostin in the aortic valves (AoV) and aortic wall (Ao). Periostin expression within the forming aortic valves seems to be widespread. (e) Image of an adult mouse heart showing intense periostin immunostaining in the pulmonary valves (PV). Periostin expression is also observed in the pulmonary wall in a loosely organized pattern (arrowheads). (f) Higher magnification view of (e), showing periostin expression in the pulmonary valve leaflets (PV). (g) Image of an adult mouse heart showing intense immunostaining of periostin in the aortic valves (AoV). Periostin is also expressed in the tendinous cords of the valve supporting apparatus (arrow). (h) Higher magnification view of (g), showing intense staining of periostin in the aortic valves and aortic wall. Note that periostin is also localized in MF20-negative non-myocardial cells in the left ventricular wall (arrowheads, green) in the ventricular myocardium. IVS, interventricular septum; LA, left atrium; LV, left ventricle; RA, right atrium; RV, right ventricle.

We extensively assessed periostin protein expression pattern in AV valvulogenesis in mice [27]. However, periostin expression in OT in later development and in the adult has not been rigorously addressed. As we previously reported, periostin protein was first detected within AV cushion mesenchyme at ED 9.5. As cushion formation progresses, intense and predominantly extracellular expression of periostin is observed in enlarging AV and OT cushions at ED 11 and ED 12.5 [27]. At ED 13.5 strong periostin expression is detected within the developing pulmonary trunk (PT) (Figure 1a, 1b), and developing pulmonary and aortic valves (Figure 1c, 1d). Expression in the developing aortic valves appears to be widespread whereas the pulmonary trunk exhibits more fibril-like immune staining. The developing epicardium (Ep) and endocardial lining of the trabeculae (arrowheads) also exhibit intense periostin staining (Figure 1a, 1c). Up to adult stage, periostin expression remains intense in the pulmonary and aortic valves (Figure 1e–1h). Periostin expression is also robust within the tendinous cords (an arrow) and aortic wall (Figure 1g, 1h). A loosely-organized periostin staining pattern is observed in the pulmonary arterial wall (Figure 1e). Periostin is also expressed in MF-20-negative nonmyocardial cells in the left ventricle of the adult mice (Figure 1h). These findings indicate that periostin is predominantly expressed in non-cardiomyocyte cells in developing hearts and intense expression of periostin remains in all four cardiac valves, i.e. aortic, pulmonary, tricuspid and mitral valves up to the adult stage in mice.

### Periostin expression is largely diminished in the aortic valves in the Smad6−/− neonatal hearts

**Figure 2:**
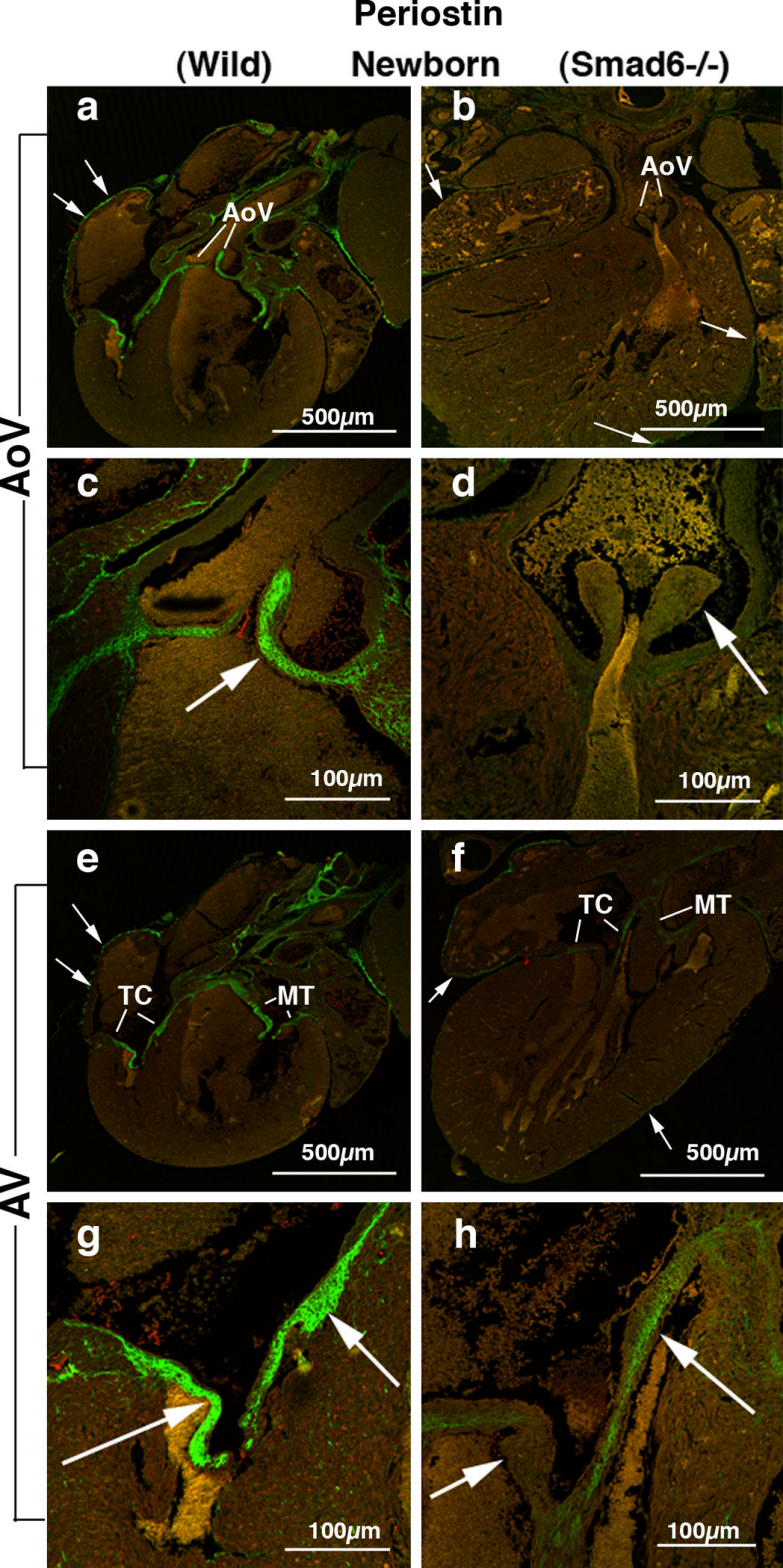
Periostin localization in Smad6+/+ (wild type) (panels a, c, e and g) and Smad6−/− littermate (panels b, d, f and h) newborn mouse aortic (AoV) and AV (AV) valves. (a) Intense periostin expression is shown in aortic valves (AoV) in a Smad6+/+ (wild type) newborn mouse heart. Arrows indicate periostin expression in the epicardium surrounding the atrial wall. (b) Periostin expression in a Smad6−/− newborn mouse heart. Periostin is localized in the epicardium (arrows); however, only weak immunostaining is observed in the aortic valves (AoV). (c) Higher magnification view of the aortic valves (arrow) in panel a, showing intense expression of periostin. (d) Higher magnification view of the aortic valves (arrow) in panel b. Periostin protein expression in the aortic valves is diminished in a Smad6−/− newborn mouse heart. (e) Intense periostin expression is shown in the tricuspid (TC) and mitral (MT) valves in a Smad6+/+ (wild type) newborn mouse heart. Arrows indicate periostin expression in the epicardium. (f) Periostin expression in a Smad6−/− newborn mouse heart. Periostin is localized in the epicardium (arrows); however, weaker immunostaining is observed in the tricuspid (TC) and mitral (MT) valves in a Smad6−/− newborn mouse heart than in the wild type heart. (g) Higher magnification view of the tricuspid valves (arrows) in panel e, showing intense expression of periostin. (h) Higher magnification view of the tricuspid valves (arrows) in panel f, showing weaker periostin immunostaining in the Smad6−/− heart than in the wild type heart.

Periostin protein expression remains intense in the OT and AV valves up to the adult stage in the wild type mice (Figure 1). General morphological defects in Smad6-mutant mice were reported by Galvin et al. [17]. We found robust periostin immunostaining in all four cardiac valves in the wild type neonatal mouse hearts (Figure 2a, 2c, 2e, 2g). In contrast, periostin expression is significantly diminished in the aortic valves in the Smad6−/− newborn mice (Figure 2b, 2d; 5A). Periostin expression is also slightly reduced in the AV valves (Figure 2f, 2h; 5A). Periostin expression is also largely diminished in the aortic wall in the Smad6−/− mice. Our quantitative evaluation of periostin immunointensity revealed that periostin expression was significantly reduced in the aortic and AV valves in Smad6−/− mice (p<0.05) (Figure 5). Reduction of periostin expression is more prominent in the aortic valves than in the AV valves.

### Versican expression is largely diminished in the aortic and AV valves in Smad6−/− neonatal hearts

**Figure 3:**
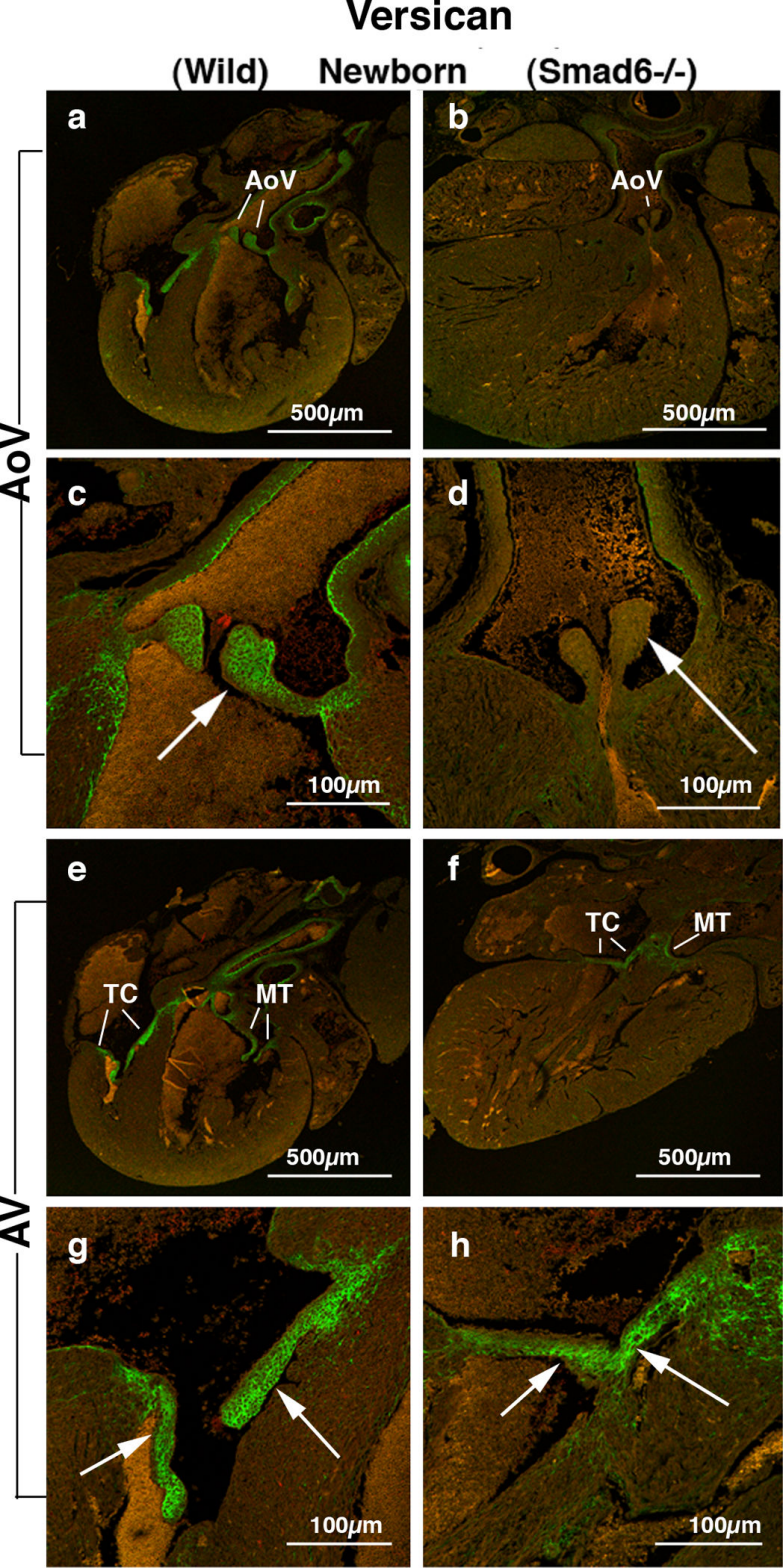
Versican localization in Smad6+/+ (wild type) (panels a, c, e and g) and Smad6−/− littermate (panels b, d, f and h) newborn mouse aortic (AoV) and AV (AV) valves. (a) Intense versican expression is seen in the aortic (Ao) valves in a Smad6+/+ (wild type) newborn mouse heart. (b) Versican expression in a Smad6−/− new-born mouse heart. Weak versican immunostaining is observed in the aortic valves (Ao). (c) Higher magnification view of the aortic valves (arrow) in panel a, showing intense expression of versican. (d) Higher magnification view of aortic valves (arrow) in panel b. Versican expression in the aortic valves is diminished in a Smad6−/− newborn mouse heart. (e) Intense versican expression is shown in tricuspid (TC) and mitral (MT) valves in a Smad6+/+ (wild type) newborn mouse heart. (f) Versican expression in a Smad6−/− newborn mouse heart. Versican is localized in tricuspid (TC) and mitral (MT) valves. (g) Higher magnification view of the tricuspid valves (arrows) in panel e, showing intense expression of versican in tricuspid valves. (h) Higher magnification view of the tricuspid valves (arrows) in panel f. Versi-can expression in the tricuspid valves is slightly reduced in a Smad6−/− newborn mouse heart.

To explore expression patterns of other critical valvulogenic ECM proteins whose expression can be altered by BMP signaling, expression of versican – a chondroitin sulfate proteoglycan (CSPG) – was evaluated in the Smad6-null and littermate wild type neonatal mouse hearts. Versican was selected because it is known to be essential for AV valvulogenesis [30]. We found robust versican immunostaining in all four cardiac valves in the wild type newborn mouse hearts (Figure 3a, 3c, 3e, 3g). In contrast, in Smad6−/− newborn mice versican expression is significantly diminished in the aortic valves (Figure 3b, 3d; 5B) and also reduced in the AV valves but to a much lesser extent (Figure 3f, 3h; 5B). Versican expression is also largely diminished in the aortic wall. Our quantitative evaluation of versican immunointensity revealed that versican expression was significantly reduced in the aortic valves in Smad6−/− mice (p<0.05) (Figure 5B). Reduction of versican expression is much more prominent in the aortic valves than in the AV valves.

We also examined the expression pattern of another CSPG, aggrecan, in the newborn hearts. The aggrecan expression pattern was examined because aggrecan expression can be increased by up regulated BMP signaling in AV valve primordial endocardial cushion cells in culture [31]. We found that aggrecan is not significantly expressed in either Smad6−/− or wild type littermate valves at birth and there was no significant difference between Smad6−/− and wild type (not shown).

### Hyaluronan (HA) deposition is reduced in the aortic valve but not significantly reduced in the AV valves of Smad6−/− newborn mouse hearts

**Figure 4:**
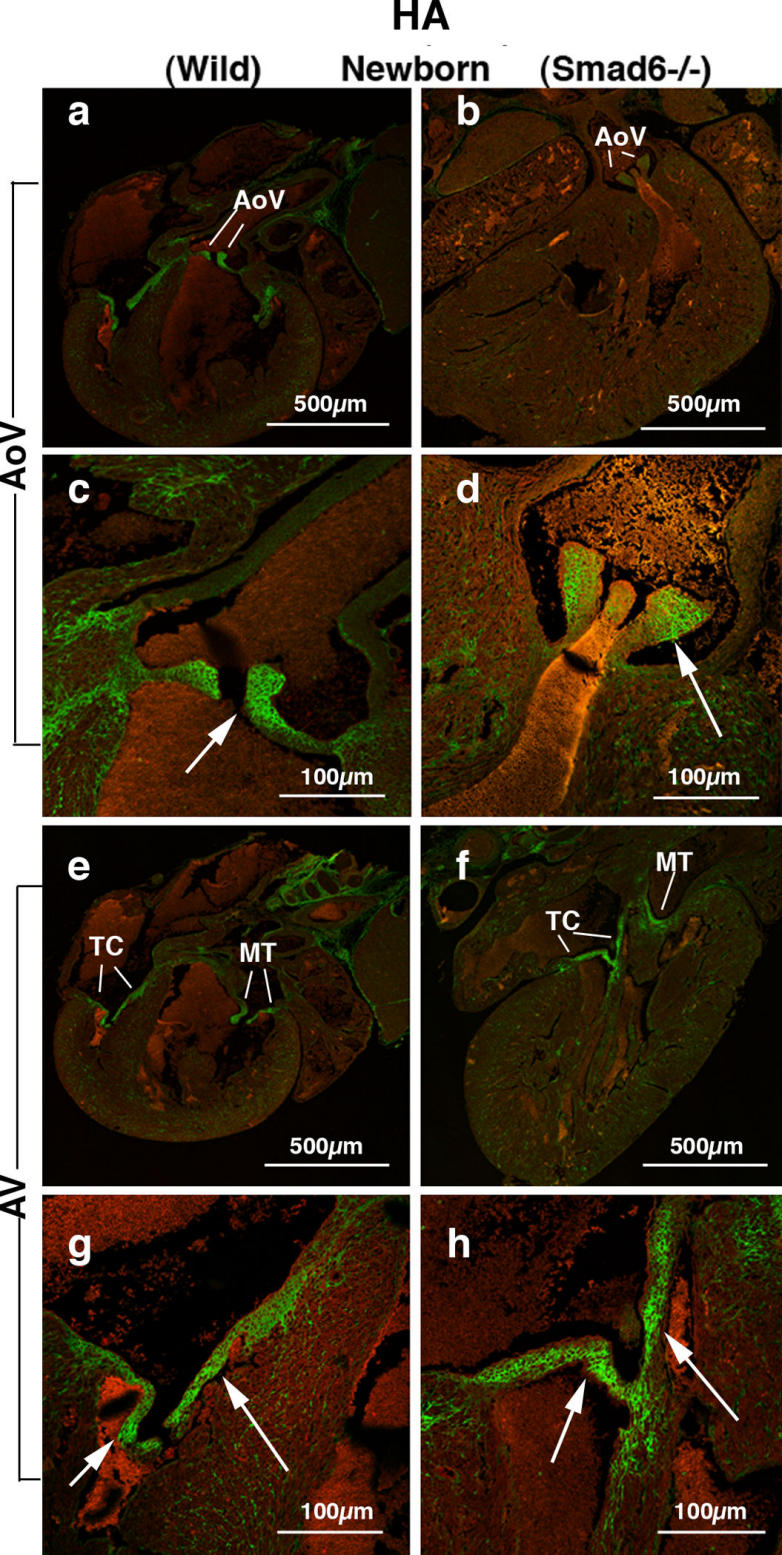
Hyaluronan (HA) deposition in Smad6+/+ (wild type) (panels a, c, e and g) and Smad6−/− littermate (panels b, d, f and h) newborn mouse aortic (AoV) and AV (AV) valves. HA localization is detected by using hyaluronan-binding protein (HABP). (a) Intense HA deposition is shown in the aortic (Ao) valves in a Smad6+/+ (wild type) newborn mouse heart. (b) HA localization in a Smad6−/− newborn mouse heart. HA immunostaining is evident but weaker in the Smad6−/− aortic valves (Ao) compared to that in the wild type aortic valves. (c) Higher magnification view of the aortic valves (arrow) in panel a, showing intense expression of HA. (d) Higher magnification view of aortic valves (arrow) in panel b. HA expression in the aortic valves is slightly reduced in a Smad6−/− newborn mouse heart. (e) Intense HA deposition is shown in tricuspid (TC) and mitral (MT) valves in a Smad6+/+ (wild type) newborn mouse heart. (f) HA localization in a Smad6−/− newborn mouse heart. HA is localized in tricuspid (TC) and mitral (MT) valves. (g) Higher magnification view of the tricuspid valves (arrows) in panel e, showing intense expression of HA in tricuspid valves (arrows). (h) Higher magnification view of the tricuspid valves (arrows) in panel f. HA deposition in a Smad6−/− tricuspid valves appears similar to that of wild type newborn mouse.

**Figure 5:**
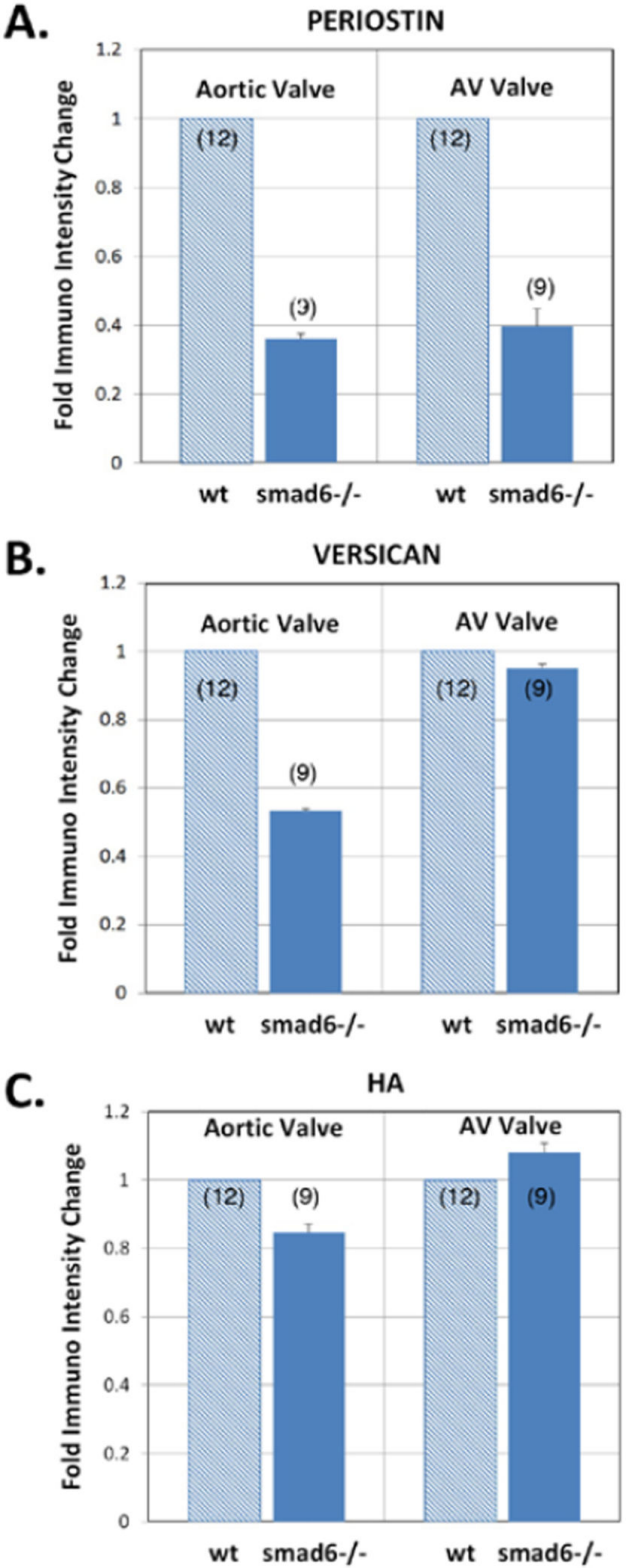
Quantitative evaluation of periostin (A), versican (B) and hyaluronan (HA) (C) immunostaining by immunointensity analysis. Immunointensity of periostin, versican in the Smad6−/− aortic valves (smad6−/−) was significantly lower than that of wild type littermate valves (wt) (p<0.01). Immunointensity of periostin in the Smad6-null AV valves was significantly lower than that of wild type littermate valves (p<0.05). Immunointensity of HA in the Smad6−/− aortic valves was also lower than that of wild type littermate valves.

To explore the expression pattern of other critical valvulogenic ECM components, HA localization was evaluated in the Smad6-null and littermate wild type newborn mouse hearts. HA was selected because it is only one known hyaluronan synthase (Has) in the heart, Has2 is known to be essential for AV EMT during valvulogenesis [32]. HA deposition was detected by using biotinylated hyaluronic acid binding protein (bHABP) as indicated in the Materials and Methods. We found intense HA immunostaining in all four cardiac valves in wild type neonatal mouse hearts (Figure 4a, 4c, 4e, 4g). HA expression is reduced in the aortic valves in Smad6−/− newborn mice (Figure 4b, 4d; 5C) but not reduced in the AV valves (Figure 4f, 4h;5C). Similarly, our quantitative evaluation of HA immunointensity revealed that HA immunostaining was reduced in the aortic valves (p<0.05) but not in the AV valves in Smad6−/− newborn mice (Figure 5C).

## Discussion

We previously reported periostin protein expression patterns specifically focusing on AV valve development and early stages of valvulogenesis [27]. Our present study describes periostin protein expression in OT in later development and in the adult. We have found in this work that periostin expression remained intense in aortic and pulmonary valves up to the adult stage. This finding is important since periostin expression in the adult stage may play significant roles in adult valve disease pathogenesis. For example, adult periostin-null mice exhibit disrupted collagen matrix layer and aortic valve calcification [33], which is also seen in some calcific aortic valve disease in human. Therefore, periostin mutant mice potentially provide a human degenerative valve disease model system. Moreover, a recent publication indicates upregulated periostin expression in vascularized valve leaflets in human atherosclerotic and rheumatic valvular heart disease (VHD) [34]. These findings suggest that periostin plays a critical role in adult cardiac valves in regulating the pathophysiology of human VHD.

Our present study shows that expression of periostin and versican is significantly reduced in aortic valves in Smad6−/− newborn mice. Smad6−/− mice, in which BMP signaling can be constitutively elevated, may provide a critical model system to study the role of upregulated-BMP signaling in valvulogenesis. Previous work has shown that periostin mRNA expression is increased in OT valve primordial cushions in an ED 14 Smad6−/− mouse [35]. Our studies with cushion mesenchymal cell cultures [15, 36] show that BMP2 and TGFβs induce periostin protein expression in culture. However, our present data indicate reduced expression of periostin in aortic valves in Smad6−/− neonatal mice, which appears to be contradictory to previously reported data. Several factors may explain this difference. Previous study in Smad6−/− mice describes periostin mRNA expression in prenatal ED 14.5 OT cushions using in situ hybridization, whereas our study presents quantitative analysis of periostin protein expression with immune localization in newborn aortic and AV valves. Thus this discrepancy could come from differences in developmental stages of the mice and/or translational problems that occurred after mRNA transcription in valve interstitial cells in Smad6−/− mice. Future studies are required to address these intriguing issues.

Up-regulation of BMP signaling by Samd6 mutation is predictable from the data obtained from cell culture assays [16]; however, secondary effects in the context of *in vivo* complexity may cause unpredictable effects on TGFβ/BMP signaling in regulating periostin and other ECM protein expression. For example, TGFβ2 knockout mice exhibit enlarged cushions at ED 14.5 and the TGFβ2 knockout phenotype suggests that TGFβ2 and BMP2 may share some common receptor(s) and Smad(s) in cardiac cushion formation *in vivo* [37]. Smad6 mutation is expected to affect Smad-dependent BMP signaling pathway. It is of interest to study if Smad independent pathways, i.e. TAK-1-dependent or MEKK3-dependent pathways [38], play any significant roles in valvulogenic ECM protein expression in Samd6 mutant mice.

Finally, since BMP signaling is known to induce BMP and activin membrane bound inhibitor (BAMBI) which reportedly inhibits TGFβ signaling [39, 40], it is of interest to further investigate whether expression of BAMBI and/or any of the TGFβ/BMP signaling pathway components are altered in Smad6-mutant mice, which would in turn down regulate periostin and other ECM protein expression in valve interstitial cells in Smad6-mutant neonatal mice.

Another intriguing issue is that aortic valves seem to be more affected than AV valves by the loss of Smad6 in regulating periostin and versican expression. Both aortic and AV cardiac valves are initially formed by transformation of endocardial cells to mesenchymal cells to form endocardial cushions. However, after formation of endocardial cushions, aortic valve primordia are populated extensively by cardiac neural crest cells [41–44] and anterior heart field derived cells [45], whereas, AV valves are predominantly derived from endocardium with Substantial contribution from the epicardial derived cells (EPDC) [46]. Smad6 is expressed in both AV and aortic valve progenitors [17, 18]. However, our data presented in this paper indicate that aortic and AV valves differentially express periostin and versican under the consequence of Smad6 mutation *in vivo*, suggesting that valve interstitial cells in the aortic and AV valves may have differential responsiveness to Smad6-mutation in regulating periostin and versican expression. Our data suggests that cell lineage/origin-dependent differential response to other signaling molecule(s) may regulate periostin and ECM protein expression in valve interstitial cells during valvulogenesis.
